# Supplementary material for: Genome-Wide Identification and Characterization of Effector Candidates with Conserved Motif in Falciphora oryzae
Source: Int J Mol Sci. 2024 Jan 4;25(1):650. doi: 10.3390/ijms25010650 (PMC10779213; doi:10.3390/ijms25010650)
Supplement: Supplementary file 1 [file ijms-25-00650-s001.zip › ijms-2751486-supplementary.pdf]

Table S1. Primers mentioned in the article.

Primer usage	Primer name	Primer sequence(5'-3')
Transient expression assay	TRV-11649-F	AAGTGGCTTTCTAGCGGATCCATGAAGTACTCCACCGCCCTCG
	TRV-11649-R	AACATCGTATGGGTATCCCGGGGTACGCAGCGAAGAAAGCCAG
	TRV-10237-F	AAAGTGGCTTTCTAGCGGATCCATGCAGTTCAAGACCGTCCTCGTCG
	TRV-10237-R	AACATCGTATGGGTATCCCGGGCTTGGTGCCAGGGAGGGGAGC
	TRV-3960-F	AAGTGGCTTTCTAGCGGATCCATGCAGTTCTCCGTTGTCGC
	TRV-3960-R	AACATCGTATGGGTACCCGGGGCAGACGGGGACCTTGAT
	TRV-11871-F	AAGTGGCTTTCTAGCGGATCCATGATTTCCAGCATGAAGGTCGC
	TRV-11871-R	AACATCGTATGGGTATCCCGGGGAGGAGGAAGACAGCAACGCC
	TRV-12356-F	AAAGTGGCTTTCTAGCGGATCCATGGCTATCATCCGCTCTTTACACC
	TRV-12356-R	AACATCGTATGGGTATCCCGGGGGCAGAGCCTGGCTGCT
qRT-PCR assay	qrt-3960-F	CGAGAACCCTAACTTCATCCAG
	qrt-3960-R	CTTGGCGATGGTGTAGGTG
	qrt-11871-F	ACATGTCGGCATTCCCC
	qrt-11871-R	CAATCCCTGACACCGTAGAG
	qrt-10237-F	CAGTTCAAGACCGTCCTCG
	qrt-10237-R	GCAGGCAAAGTCAAAGACG
	qrt-11649-F	GATTGTCTCTTCAGGAACCGG
	qrt-11649-R	TGGTACCGGGCATAAAAGTG
	qrt-12356-F	CGCGACAGCCACCAGCTG
	qrt-12356-R	AGACCGTGGCTTGGCGGC
	qrt-Tubulin-F	CTGTCAACGACCCTTTCA
	qrt-Tubulin-R	CTGTCAACGACCCTTTCA

Table S2. Effector candidates containing identical sequence of RXLR.

Type	Gene accession	RXLR feature	Conserved domain (pfam)	EffectorP prediction	Effector probability	WT FPKM	2dpi FPKM	6dpi FPKM	15dpi FPKM
Secreted proteins containing identical sequence of RXLR	g10077	RDLR	N.A.	Apoplastic effector	0.59	0.5	2.1	1.9	1.5

Table S3. Effector candidates containing complete RXLR-dEER-like motif.

Type	Gene accession	RXLR-dEER feature	Conserved domain (pfam)	EffectorP prediction	Effector probability	WT FPKM	2dpi FPKM	6dpi FPKM	15dpi FPKM
Secreted proteins containing a complete sequence of RXLR-dEER-like	g938	KGLL-HDDK	Cyclophilin type peptidyl-prolyl cis-trans isomerase(PF00160)	Cytoplasmic effector	0.546	80.9	443.7	519.7	348.1
	g7747	KYYR-GDEK	N.A.	Apoplastic effector	0.734	0.1	2.8	23.2	8.7
	g7112	KSLA-AEER	N.A.	Cytoplasmic effector	0.669	3.3	67.0	31.2	33.8
	g6636	KYYR-GDEK	N2,N2-dimethylguanosine tRNA methyltransferase(PF02005)	Apoplastic effector	0.583	24.5	81.8	279.2	128.5
	g5512	KKKG-AEDK	Fungal kinase associated-1 domain(PF16797)	Cytoplasmic effector	0.828	2.2	330.6	295.9	209.6
	g3870	KAKR-NDDK	N.A.	Cytoplasmic effector	0.665	0.9	28.1	4.5	11.2
	g3164	RWMA-PDEK	N.A.	Cytoplasmic effector	0.803	0.2	281.0	351.8	211.0
	g2481	KGKA-LEER	Collagen triple helix repeat(PF01391)	Cytoplasmic effector	0.839	0.1	0.3	1.2	0.5
	g2119	RTYT-GEDK	N.A.	Cytoplasmic effector	0.544	4.3	9.9	38.6	17.6
	g2095	KRLT-PEEK	N.A.	Cytoplasmic effector	0.674	1.3	7.2	5.2	4.6
	g1368	RVKA-GDER	Pectinesterase(PF01095)	Apoplastic effector	0.583	4.5	13.4	43.1	20.3
	g13062	KIMR-DDDR	N.A.	Apoplastic effector	0.893	22.4	36.0	47.8	35.4
	g12291	KDKA-KDDK	E4 protein(PF02711)	Cytoplasmic effector	0.524	11.8	41.4	31.5	28.2
	g12285	KDFA-ADDK	N.A.	Cytoplasmic effector	0.658	1.2	33.5	82.2	39.0
	g11908	RDLG-DDEK	LicD family(PF04991)	Cytoplasmic effector	0.62	9.4	34.8	36.3	26.8
	g1156	HVLA-KEEK	Heat-labile enterotoxin alpha	Cytoplasmic effector	0.776	2.4	5.7	5.3	4.5

		chain(PF01375)						
g10438	RLYL-SDDR	Glycosyl hydrolase family 7(PF00840)	Apoplastic effector	0.639	0.1	5.3	167.5	57.6
g10339	KNKR-EEEK	N.A.	Cytoplasmic effector	0.588	6.6	71.7	71.6	50.0
g10282	KGIL-SEDK	N.A.	Apoplastic effector	0.598	0.8	4.9	9.6	5.1
g10224	KCYT-FEDR	N.A.	Apoplastic effector	0.924	3.2	7.1	7.9	6.0
g10077	RDLR-SDER	N.A.	Apoplastic effector	0.59	0.5	2.1	1.9	1.5
g10066	KWKA-DEDK	N.A.	Cytoplasmic effector	0.654	3.8	8.5	10.7	7.7
g3869	KQLA-KEDK	Mannosyltransferase(PF11051)	Cytoplasmic effector	0.745	9.7	48.6	33.2	19.1
g3691	KVFR-ADDK	Thioredoxin(PF00085)	Cytoplasmic effector	0.68	169.5	380.5	508.5	335.6
g2686	KFFL-IEDR	N.A.	Cytoplasmic effector	0.704	135.4	535.0	452.5	152.3
g1968	RVFL-VEER	Cutinase (PF01083)	Apoplastic effector	0.667	7.9	31.1	40.4	14.8
g1935	KDWL-SEDK	Ish1 (PF10281)	Cytoplasmic effector	0.563	4.5	32.9	18.2	16.3
g13036	HMKA-EEEK	Peptidase family M28(PF04389)	Cytoplasmic effector	0.556	74.4	230.7	188.0	120.5

Table S4. Effector candidates containing Y/F/WxC motif

Gene accession	Y/F/WxC feature	Conserved domain (pfam)	EffectorP prediction	Effector probability	WT FPKM	2dpi FPKM	6dpi FPKM	15dpi FPKM
g5612	YPC	Family of unknown function (PF20238)	Apoplastic effector	0.592	784.5	1745.5	5178.2	2569.4
g4041	FIC	N.A.	Cytoplasmic effector	0.78	6.8	11.2	13.8	10.6
g3965	YVC	N.A.	Apoplastic effector	0.74	0.3	162.7	230.2	131.1
g3525	WDC	Glycosyl hydrolase family 45(PF02015)	Apoplastic effector	0.839	0.9	9.8	461.7	157.4
g3166	YGC	Uncharacterized protein conserved in bacteria (PF09937)	Cytoplasmic effector	0.862	0.6	24.7	30.6	18.6
g3164	WTC	N.A.	Cytoplasmic effector	0.803	0.2	281.0	351.8	211.0
g2691	FTC	Glucosidase II beta subunit-like(PF12999)	Cytoplasmic effector	0.504	28.5	145.4	115.6	96.5
g2444	FYC	N.A.	Apoplastic effector	0.814	12.5	28.6	126.1	55.7
g14270	YQC	ToxB N-terminal domain(PF18224)	Cytoplasmic effector	0.581	0.8	8.4	11.2	6.8
g12983	FKC	N.A.	Apoplastic effector	0.574	17.0	64.7	35.0	38.9
g12957	WEC	N.A.	Apoplastic effector	0.789	1.1	26.8	5.4	11.1
g12852	FVC	Alternaria alternata allergen 1(PF16541)	Apoplastic effector	0.658	1.7	256.9	311.0	189.9
g12181	YTC	Cellulase (glycosyl hydrolase family 5) (PF00150)	Apoplastic effector	0.509	10.8	74.7	211.3	98.9
g121	WDC	Glycosyl hydrolase family 45(PF02015)	Apoplastic effector	0.896	3.3	8.5	32.2	14.7
g11545	YDC	N.A.	Apoplastic effector	0.831	1.3	5.1	5.8	4.1
g10237	FAC	CFEM domain (PF05730)	Apoplastic effector	0.829	12.1	1128.3	2355.9	1165.4
g10077	YGC	Domain of unknown function (PF16129)	Apoplastic effector	0.59	0.5	2.1	1.9	1.5
g11297	YGC	N.A.	Cytoplasmic effector	0.804	1.5	16.9	282.6	106.1

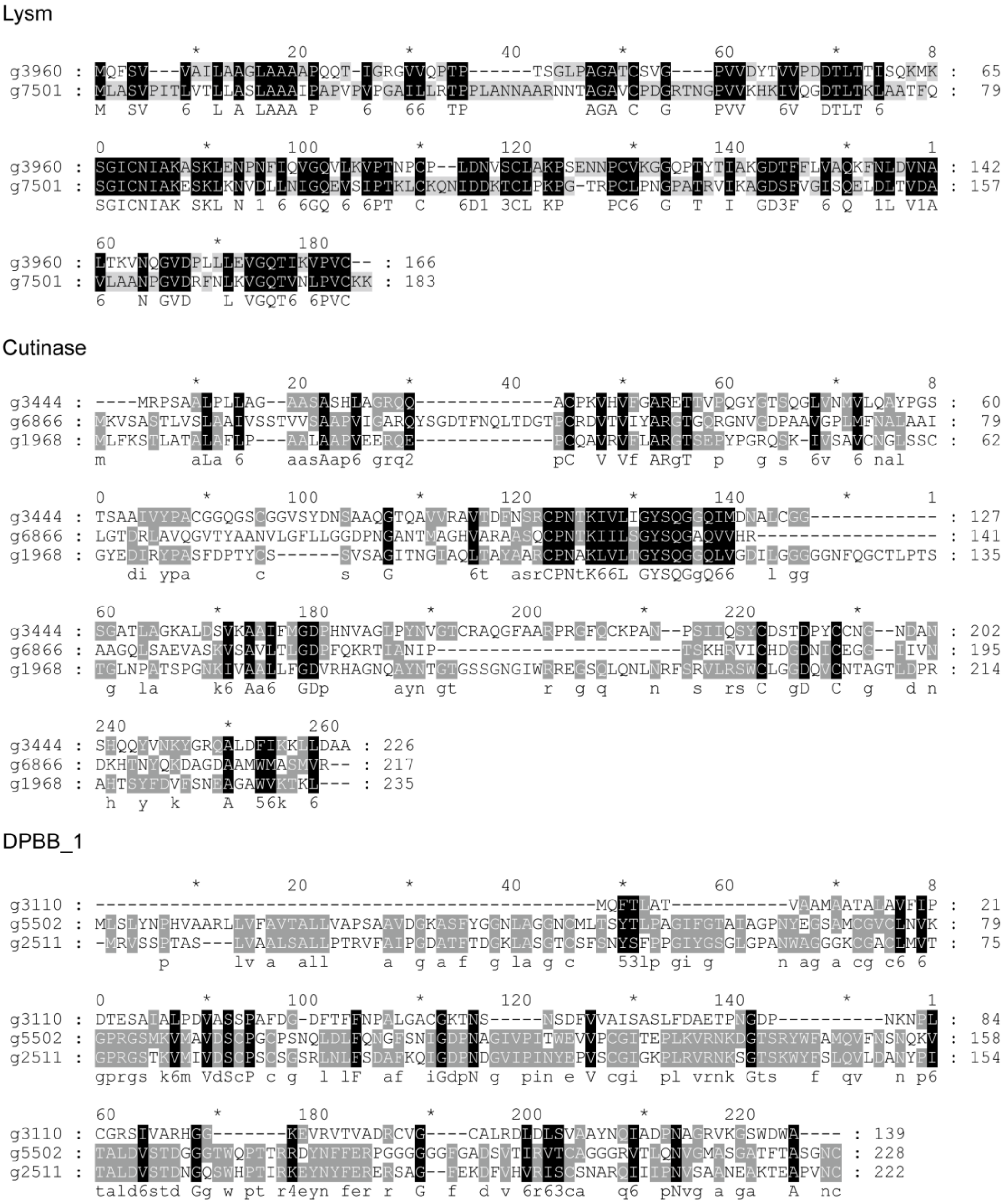

Figure S1. Multiple sequence alignment of *F.oryzae* effector candidates containing Lysm、DPBB_1 and Cutinas domains. The full sequences of the proteins were aligned by Clustal X. The intensity of black shadow reflects the identity of amino acids at each position.
